# Modelling the cost-effectiveness of a rapid diagnostic test (IgMFA) for uncomplicated typhoid fever in Cambodia

**DOI:** 10.1371/journal.pntd.0006961

**Published:** 2018-11-19

**Authors:** Mari Kajiwara Saito, Christopher M. Parry, Shunmay Yeung

**Affiliations:** 1 Department of Non-Communicable Disease Epidemiology, Faculty of Epidemiology and Population Health, London School of Hygiene and Tropical Medicine, London, United Kingdom; 2 Clinical Sciences, Liverpool School of Tropical Medicine, Liverpool, United Kingdom; 3 School of Tropical Medicine and Global Health, Nagasaki University, Nagasaki, Japan; 4 Department of Global Health and Development, Faculty of Public Health and Policy, London School of Hygiene and Tropical Medicine, London, United Kingdom; 5 Department of Clinical Research, Faculty of Infectious and Tropical Disease, London School of Hygiene and Tropical Medicine, London, United Kingdom; McGill University, CANADA

## Abstract

Typhoid fever is a common cause of fever in Cambodian children but diagnosis and treatment are usually presumptive owing to the lack of quick and accurate tests at an initial consultation. This study aimed to evaluate the cost-effectiveness of using a rapid diagnostic test (RDT) for typhoid fever diagnosis, an immunoglobulin M lateral flow assay (IgMFA), in a remote health centre setting in Cambodia from a healthcare provider perspective. A cost-effectiveness analysis (CEA) with decision analytic modelling was conducted. We constructed a decision tree model comparing the IgMFA versus clinical diagnosis in a hypothetical cohort with 1000 children in each arm. The costs included direct medical costs only. The eligibility was children (≤14 years old) with fever. Time horizon was day seven from the initial consultation. The number of treatment success in typhoid fever cases was the primary health outcome. The number of correctly diagnosed typhoid fever cases (true-positives) was the intermediate health outcome. We obtained the incremental cost effectiveness ratio (ICER), expressed as the difference in costs divided by the difference in the number of treatment success between the two arms. Sensitivity analyses were conducted. The IgMFA detected 5.87 more true-positives than the clinical diagnosis (38.45 versus 32.59) per 1000 children and there were 3.61 more treatment successes (46.78 versus 43.17). The incremental cost of the IgMFA was estimated at $5700; therefore, the ICER to have one additional treatment success was estimated to be $1579. The key drivers for the ICER were the relative sensitivity of IgMFA versus clinical diagnosis, the cost of IgMFA, and the prevalence of typhoid fever or multi-drug resistant strains. The IgMFA was more costly but more effective than the clinical diagnosis in the base-case analysis. An IgMFA could be more cost-effective than the base-case if the sensitivity of IgMFA was higher or cost lower. Decision makers may use a willingness-to-pay threshold that considers the additional cost of hospitalisation for treatment failures.

## Author summary

Typhoid fever is a common disease among children in Cambodia. It can be fatal or lead to chronic faecal carriage if not treated. In resource-limited settings, typhoid fever is often diagnosed and treated presumptively. This study evaluated the cost-effectiveness of introducing a rapid diagnostic test for typhoid fever in a remote setting in Cambodia. In a hypothetical cohort with 1000 children in each arm we compared the use of a rapid diagnostic test (RDT) with a presumptive clinical diagnosis. In each arm, we calculated the number of true-positive typhoid fever cases detected, treatment success at seven days, and the cost of making a correct diagnosis and providing the correct treatment. The RDT detected 5.87 more true positives, had 3.61 more treatment successful cases, but the total cost was $ 5700 higher per 1000 children. Additional analysis showed that the RDT would be more cost-effective if the sensitivity could be improved or cost lowered.

## Introduction

Typhoid fever is estimated to cause 21 million new cases per year worldwide [1, 2]. It is a common disease among children in resource-limited settings such as Cambodia [2–5]. In Cambodia, which is classified as a high incidence area for typhoid fever, the distribution of typhoid fever cases is highest in children aged under 15 years [6]. Typhoid fever is a systemic infection with non-specific clinical features that make it difficult to differentiate from other common febrile illnesses [5, 7]. A dry cough, for example, is a common symptom and may lead to confusion with pneumonia [5, 7]. As many as ten to fifteen percent of the patients who have been sick for more than two weeks with typhoid develop severe complications (gastrointestinal haemorrhage, shock or hepatitis) [1, 5, 8]. The case fatality ratio was reported to be 10–30% in the pre-antimicrobial era [1, 9]. Effective antimicrobial treatment should decrease the case fatality ratio to less than 1%. Prompt diagnosis and appropriate antimicrobial drug therapy is needed to avoid severe or fatal disease, relapse and also acute and chronic faecal carriage that may lead to onward transmission of typhoid [9]. Empirical treatment with antimicrobials should be guided by local data since the susceptibility of isolates widely varies among countries and regions [10, 11]. *Salmonella enterica* serovar Typhi (*S*. Typhi) isolates at Angkor Hospital for Children (AHC) in Siem Reap, north-west Cambodia are dominated by strains that are multi-drug resistant (MDR) (resistant to chloramphenicol, ampicillin and co-trimoxazole) and with decreased susceptibility to ciprofloxacin [12]. In this setting, oral azithromycin remains an option for the initial empiric treatment of children with suspected uncomplicated typhoid fever. Children with a febrile illness in this setting can have other bacterial illnesses such as community-acquired pneumonia [13, 14]. The common causative organisms among children are *Streptococcus pneumoniae*, *Haemophilus influenzae* type b, and following the guidelines of the World Health Organization, they can be treated with amoxicillin [15]. Amoxicillin would be inadequate in this area if the true diagnosis was MDR typhoid [12].

Differentiating typhoid from other causes of fever in children in this area is challenging without rapid and reliable diagnostic tests. The recommended reference standard diagnostic tests for typhoid fever are blood culture or bone marrow culture with sensitivities of 40–80% and 80–95% respectively [5, 7]. Both tests are invasive, technically demanding and are not available in remote health care settings where the majority of uncomplicated typhoid cases present. Additionally, they require several days for a positive result to be confirmed [4, 7]. Low-cost diagnostic test, such as Widal test, is still widely used but lacks sensitivity and specificity [7, 16].

There are a number of commercially available rapid diagnostic tests (RDTs) for typhoid fever [17]. For example, an immunoglobulin M lateral flow assay (IgMFA), which detects immunoglobulin M (IgM) antibodies against the lipopolysaccharide of *S*. Typhi, has been evaluated in Cambodian and Bangladeshi children [16, 18]. Among other RDTs, this IgMFA has advantages of simplicity to perform, no need for refrigeration, and giving results within 15–30 minutes. It has a sensitivity of 59% and specificity of 98% [18]. The availability of such an RDT in resource-limited settings might be expected to lead to an early and appropriate choice of antimicrobial drug treatment.

The overall costs of typhoid fever have been evaluated in studies in India and other Southeast Asian countries, but a cost-effectiveness analysis of the use of RDTs in resource-limited settings has not been performed [19, 20]. This study aimed to evaluate the effectiveness and costs of using the IgMFA in a typhoid fever endemic country and to inform decision-making on whether to introduce the IgMFA in a remote health centre setting in Cambodia. Cambodia was chosen because it is a high-burden country for typhoid and because of the availability of data to inform model parameterization. In rural Cambodia half of the population is estimated to be aged under 25 years [21] and in children presenting to health facilities with fever, the prevalence of uncomplicated *S*. Typhi infection is estimated to be between 4.5% and 9.0% [18, 22, 23].

## Methods

We conducted a cost-effectiveness analysis (CEA) of using an RDT for typhoid fever diagnosis, IgMFA, in a remote health centre setting in Cambodia from a healthcare provider perspective. The comparator was the current standard of care which is presumptive clinical diagnosis without an RDT. The costs that were included were therefore the direct costs of the diagnostic test (including supply, labour and equipment costs) and the costs of treatment. Costs borne by patients including indirect costs due to productivity loss were not included. The effects were natural units: treatment success at 7 days and an intermediate outcome of correctly diagnosed typhoid fever. Cost-utility analysis presenting disability-adjusted life years (DALYs) was not chosen since it would not provide understanding of the net effects of offering a new diagnostic test.

### Model choice and descriptions

Existing models for malaria RDT and a model for diagnosis of sepsis in low-resource settings were modified to construct a new decision analytic model for typhoid fever [24–26].

Two diagnostic approaches were compared. The new intervention arm employed the IgMFA and the comparator arm was a presumptive clinical diagnosis based on patient’s symptoms. The performance of the RDT was based on studies of the Life Assay Test-It IgMFA (Life Assay Diagnostics, Cape Town, South Africa) in Cambodia [16, 18]. Clinical diagnosis was made based on current clinical practice in AHC and other hospitals [16, 18, 27]. A patient was considered to have typhoid fever if febrile for more than 3 days and one or more of four clinical features was present; presence of abdominal symptoms (constipation, diarrhoea or abdominal pain); body temperature >39 °C; hepatomegaly and/or splenomegaly; or no alternative confirmed diagnosis established.

**Fig 1:**
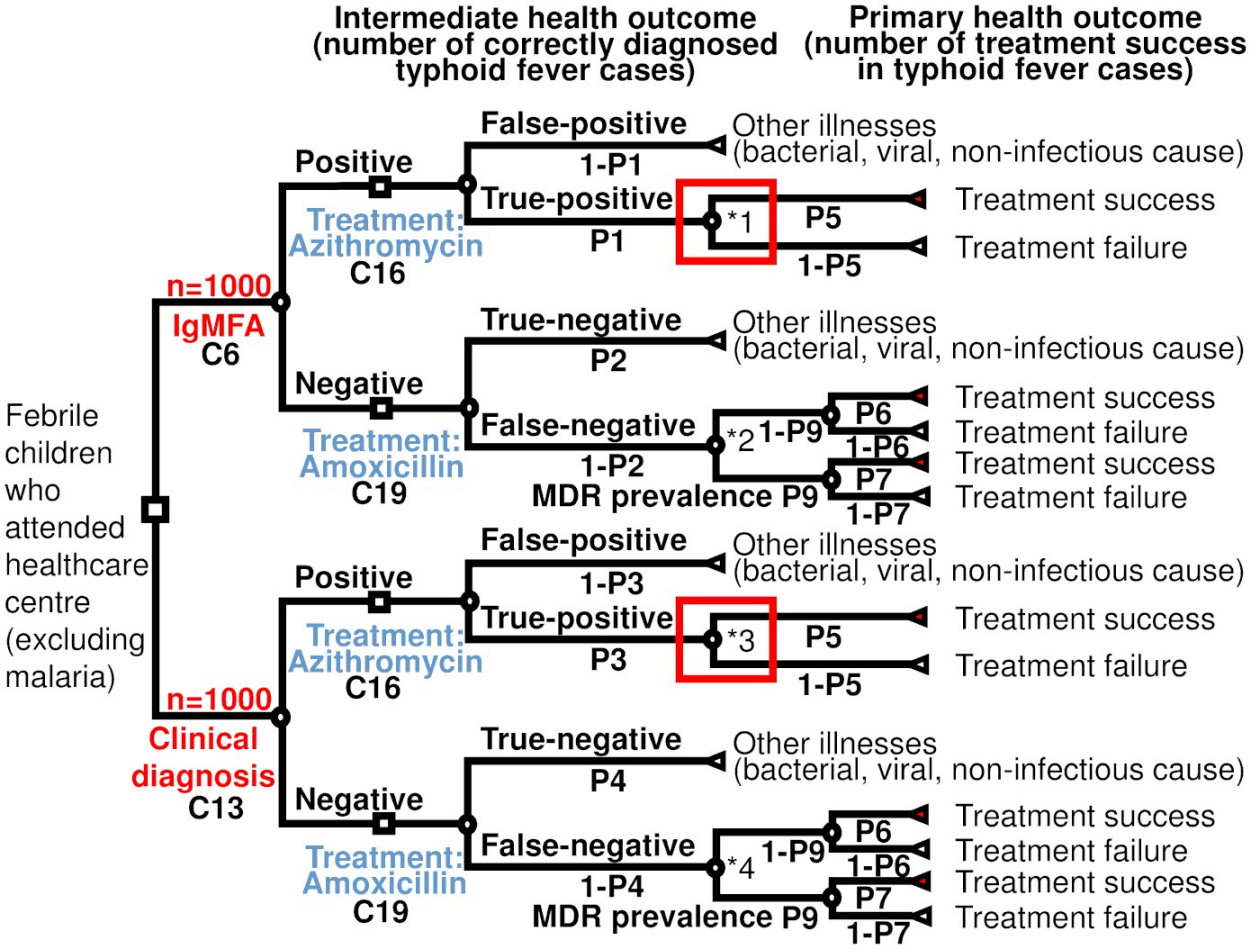
Decision tree model for diagnosis and treatment for typhoid fever. * MDR strains exist equally among true-positives and false-negatives. Since probability of treatment success in azithromycin is the same between MDR strains and non-MDR strains, *1 and *3 are not branched to P9 and (1-P9).

A decision tree model was constructed with a hypothetical cohort of 1000 children using Microsoft Excel (2016). We hypothesised that the children aged from 0 to 14 years, who visited a health centre in Siem Reap province, Cambodia, with undifferentiated fever, were eligible for the entry in this model. Malarial patients were excluded from the beginning of this model assuming that malaria was tested for and excluded before suspecting typhoid fever. Patients who had complicated symptoms, such as shock, encephalopathy, convulsions, bleeding, deep jaundice or suspected gut perforation were excluded as they would be referred to hospital. The decision tree model is shown in Fig 1.

Following the result of a diagnostic test, two treatment choices were defined based on literature [15, 27–35]. When a test was positive for typhoid fever, azithromycin (250 mg per day) for five days was prescribed. When a test was negative, amoxicillin (1500 mg per day) for five days was prescribed. The average weight of 15 kg was estimated based on data on the age distribution of children attending outpatient appointments in Cambodia [36] (S1 Appendix).

### Selection of health outcomes

The effect of diagnosis was evaluated as two health outcomes. The number of correctly diagnosed typhoid fever cases (i.e. true-positives) was set as the intermediate health outcome and the number of treatment success for typhoid fever at day seven was set as the primary health outcome. The intermediate model outputs included number and cost of treatment of false-positives and true-negatives. Another choice of health outcome could be the number of correctly diagnosed cases (i.e. both true-positives and true-negatives). However, the main issue for diagnosis and treatment of typhoid fever in remote setting where MDR typhoid is common and where amoxicillin would not be effective is missing true-positives (under-diagnosis and inadequate treatment). Ineffective treatment of the missed case of typhoid fever may lead to the development of complications, hospital admission and mortality. Therefore, in this study, health outcome measures used in the cost-effectiveness analysis focused on the number of true-positives only and the number of treatment success in typhoid fever cases. Health outcomes were calculated using prevalence of typhoid fever, sensitivity and specificity of diagnostic tests derived from studies in Siem Reap province, Cambodia (S2 Appendix).

### Model assumptions

Several assumptions were made for the decision tree model. In rural areas, 18% of the people seek initial treatment at health centres run by the public sector, followed by private clinics (16%) and pharmacies (8%) [21]. For the purposes of the model we assumed that no child had been treated with antimicrobial drugs before visiting the health centre. A febrile child was assumed to have only one disease and co-infection was not considered. Regarding treatment, we assumed that the health centre workers perfectly adhered to the results of the tests and the treatment protocols in this model, the availabilities of both azithromycin and amoxicillin were 100%, and the patients’ adherence to the treatments was 100%. Although adherence issues are critically important to consider prior to implementation, there were no or limited local data on these parameters. As an important first step, this study aims to explore whether the introduction of IgMFA has the potential to be cost-effective.

We also assumed that there were no azithromycin-resistant strains of *S*. Typhi in the area. Thus, the MDR strains had the same treatment success probability as that of non-MDR strains when treated with azithromycin (Fig 1 *). On the contrary, we assumed that the MDR strains had 0% treatment effect if treated with amoxicillin. There are no available data for the prevalence of the MDR strains in the community. However, we set the MDR prevalence in the community at 50% based on the assumption that the prevalence of MDR is lower than that in the hospital settings in Siem Reap at 85% [12].

### Time horizon

The primary health outcome was measured as the number of children with treatment success at day seven from the start of treatment in typhoid fever cases. The number of children with treatment success at day seven could be a useful measure of diagnosis since it represents the effect of correct diagnosis and subsequent treatment choice and is commonly used for assessing efficacy [32]. It is clinically and biologically plausible to think that after the seventh day patients will seek hospital care if they have no improvement in clinical symptoms.

In all arms, we assumed that no additional diagnostic tests would be performed until the endpoint of this model on day seven. If the diagnostic test for typhoid fever was negative, other possibilities were considered and amoxicillin was used presumptively.

### Parameters

Parameter inputs for the model were obtained from published literature from AHC and other literature from Cambodia or Laos whose study setting is similar to that in Siem Reap, Cambodia [13, 14, 16, 18, 23, 37], and are shown in Table 1. The reference test for both IgMFA and clinical diagnosis was blood culture [18]. Prevalence was estimated from typhoid fever cases confirmed by blood culture test based on local hospital data [18]. Supplementary literature reviews were also used to cross-check the values. The probabilities of treatment success with azithromycin and amoxicillin were calculated from literature of randomised controlled trials [27–35]. For synthesising the effect, the weighted average assuming random effects was calculated using Stata MP 14.1 [38] (S3 Appendix).

**Table 1:** Sensitivity and specificity of diagnostic tests, probabilities of treatment success and epidemiology of diseases.

Parameter		Value (95% CI)	Source
**Sensitivity and specificity of diagnostic tests**			
**P1**	Sensitivity of IgMFA	59% (42–77%)	[18]
**P2**	Specificity of IgMFA	98% (97–99%)	[18]
**P3**	Sensitivity of clinical diagnosis	50% (33–67%)	[18]
**P4**	Specificity of clinical diagnosis	86% (83–89%)	[18]
**Probability of treatment success in typhoid fever cases**			
**P5**	Probability of treatment success in azithromycin treated patients (same in MDR and non-MDR)	97% (91–99%)	[27–31, 33–35, 39]
**P6**	Probability of treatment success in amoxicillin treated patients (non-MDR)	71% (56–82%)	[40, 41]
**P7**	Probability of treatment success in amoxicillin treated patients (MDR)	0%	Model assumption
**Epidemiology of diseases**			
**P8**	Prevalence of uncomplicated typhoid fever (among febrile children who visited a health centre)	6.5%* (4.5%–9.0%)	[18, 22, 23]
**P9**	Prevalence of MDR strains (among typhoid fever cases diagnosed in a health centre)	50%	Model assumption [12]

95% CI, 95% confidence interval; IgMFA, Immunoglobulin M lateral flow assay; MDR, multi-drug resistant; RCT, randomised controlled trial. P8 Value* This prevalence is the prevalence in a population where malaria (2%) has been already excluded.

### Estimation of costs

Cost parameter inputs are shown in Table 2. No additional data collection was performed for this study. Direct medical costs are presented by the economic costs of diagnosis and treatment. The economic costs include recurrent costs of diagnosis (i.e. unit cost of performing a test) and treatment (i.e. unit cost of antimicrobials and supply costs). Costs of diagnosis include costs of equipment, consumables and staff salary. Starting-up costs, such as costs of training staff were also evaluated. Fixed costs such as facility costs were not evaluated in this study since the health centre facility was assumed to be already present and the facility costs would be the same for both IgMFA and the clinical diagnosis arm. Unit price data were obtained from CHOosing Interventions that are Cost Effective (WHO-CHOICE), International Drug Price Indicator Guide 2015, other literature, websites or expert opinions [18, 42–45]. The costs were derived using a micro-costing method with bottom-up approach [46]. To calculate staff costs and training costs, we assumed the personnel time on the basis of literature on malaria RDT and expert opinion [26, 47]. Regarding the costs of drugs, the median costs were derived from supplier costs of drugs and the shipping (i.e. supply) cost was set at 10%, following the recommendation of International Drug Price Indicator Guide 2015 [43] (S4 Appendix). All costs were presented in the United States (US) dollars in the year of 2016, adjusted using the World Bank purchasing power parities (PPPs) and the World Bank consumer price index (CPI) [48, 49].

**Table 2:** Cost parameters in base-case analysis (presented in US$, 2016).

Cost parameter		Value	Source
**IgMFA**			
**C1**	IgMFA test kit	$3.58	Primary data from an expert at AHC including supply cost (10%) [43].
**C2**	Consumables	$0.01	Gloves [42].
**C3**	Equipment	$0.00 (< $0.001)	Thermometer [42].
**C4**	Staff	$4.30	Assumption [45]. Staff hourly wage is $8.60 (8 hours working/day, level 2 jobs) in East Asia and Pacific. Assumed time for diagnostic procedure is 30 minutes (including counselling and drug prescribing time).
**C5**	Overhead	$0.00 (< $0.001)	Assumption [45]. No refrigerator needed.
**C6**	Total cost per test	$7.89	Sum of C1 to C5.
**C7**	Training costs (per person/ year)	$21.51	Assumption based on malaria RDT training time of 150 minutes per year (WHO) [26].
**Clinical diagnosis**			
**C8**	Test kit	$0.00	No test kit.
**C9**	Consumables	$0.00	No consumables.
**C10**	Equipment	$0.00 (< $0.001)	Thermometer [42].
**C11**	Staff	$2.15	See C4. Assumed time for diagnostic procedure is 15 minutes (including counselling and drug prescribing time) [45].
**C12**	Overhead	$0.00 (<$0.001)	Assumption. Maintenance of check list sheet.
**C13**	Total cost per test	$2.15	Sum of C8 to C12.
**Azithromycin**			
**C14**	Azithromycin (250 mg/day)	$0.181 (min $0.093, max $0.574)	Median cost of 12 supplier prices [43]. Includes 10% of supply cost.
**C15**	Duration of treatment	5 days (min 3, max 7 days)	Assumption [27–35].
**C16**	Total costs per treatment	$0.903	C14*C15.
**Amoxicillin**			
**C17**	Amoxicillin (1500 mg/day)	$0.115 (min $0.063, max $1.236)	Dose of 1500mg based on average weight of study population (15kg). Median cost of 40 supplier prices [43]. Includes 10% of supply cost.
**C18**	Duration of treatment	5 days (fixed)	[15]
**C19**	Total costs per treatment	$0.558	C17*C18.

95% CI, 95% confidence interval; AHC, Angkor Hospital for Children; IgMFA, Immunoglobulin M lateral flow assay; RDT, rapid diagnostic test; WHO, World Health Organization.

### Effectiveness measure

Other costs and effects included the number of over-treated patients, cost per child (cost-effectiveness ratio C/E), cost per correctly diagnosed and cost per treatment success in each arm.

The incremental cost-effectiveness ratio (ICER) comparing IgMFA and clinical diagnosis was calculated by measuring the difference in costs and effects and represents the additional cost to gain one additional health outcome. The ICER for both primary and intermediate health outcomes were calculated. The ICER for primary health outcome was calculated as the difference in the total costs between IgMFA and clinical diagnosis, divided by the difference in the number of treatment success between IgMFA and clinical diagnosis. The ICER for the intermediate health outcome was calculated as the difference in the total costs between two arms, divided by the difference in the number of correctly diagnosed typhoid fever cases between the two arms.

For the ICER to gain one additional treatment success, sensitivity analyses were performed. To determine which key parameter drives the results in this model (i.e. assessment of parameter uncertainty), one-way sensitivity analyses were conducted changing one variable and keeping other variables constant. We also conducted two-way sensitivity analyses to evaluate distributions of the ICER, estimated by changes in product profiles of IgMFA. Whether the ICER reaches a certain willingness-to-pay (WTP) threshold was also evaluated in the two-way sensitivity analyses. Probabilistic sensitivity analysis (PSA) was performed to address parameter and model uncertainty.

Regarding test performance, sensitivity and specificity of both IgMFA and clinical diagnosis were changed ranging from a lower to an upper 95% confidence interval (CI) value. Also, we conducted best-case scenario analyses of the sensitivity and specificity for IgMFA at 100% for each, to determine whether the ICER reaches a WTP threshold. For the clinical diagnosis, worst-case scenario analyses with 0% sensitivity and specificity for each were conducted. Concerning treatment effects, the probability of treatment success was changed from a lower to an upper 95% CI in each drug.

Regarding cost parameters, the cost for IgMFA was changed assuming that it would vary from a half to twice the price in the primary data, by applying an existing model in a diagnostic test for sepsis [26]. Cost of azithromycin and amoxicillin was changed from minimum to maximum value in each. Prevalence of typhoid fever was also changed from a lower to an upper 95% CI value. Prevalence of MDR strains was changed from 25% to 90%. Plausible range of the prevalence in MDR strains was assumed based on data in AHC [12].

A PSA was performed with Monte-Carlo simulations by simultaneously varying the variables mentioned above. The assumptions of the distribution of each variable are shown in Table 3. Health outcomes and costs were stochastically generated by 1000 simulations.

**Table 3:** Parameters changed in probabilistic sensitivity analysis (presented in US$, 2016).

Parameter		Value	Distribution	Source
**Sensitivity and specificity of diagnostic tests**				
**P1**	Sensitivity of IgMFA	59%	Beta (α = 19, β = 13)	[18]
**P2**	Specificity of IgMFA	98%	Beta (α = 446, β = 10)	[18]
**P3**	Sensitivity of clinical diagnosis	50%	Beta (α = 16, β = 16)	[18]
**P4**	Specificity of clinical diagnosis	86%	Beta (α = 404, β = 64)	[18]
**Probability of treatment success in typhoid fever cases**				
**P5**	Probability of treatment success in azithromycin treated patients (for both MDR and non-MDR)	97%	Triangular (mode = 0.97, min = 0.82, max = 1.0)	[27–31, 33–35]
**P6**	Probability of treatment success in amoxicillin treated patients (non-MDR)	71%	Triangular (mode = 0.71, min = 0.68, max = 0.75)	[40, 41]
**P7**	Probability of treatment success in Amoxicillin treated patients (MDR)	0%	Fixed	Model assumption
**Epidemiology of diseases**				
**P8**	Prevalence of typhoid fever	6.5%	Beta (α = 32, β = 459)	[18, 22, 23]
**P9**	Prevalence of MDR strains in *S*. Typhi	50%	Triangular (mode = 0.5, min = 0.25, max = 0.9)	Model assumption
**Cost of diagnosis**				
**C1**	Cost of IgMFA (including 10% supply cost)	$3.58	Triangular (mode = 3.59, min = 1.79, max = 7.15)	Model assumption (min = base*0.5, max = base*2) [26].
**C4/C11**	Salary (per hour)	$8.60	Triangular (mode = 8.60, min = 4.77, max = 11.85)	[45]
**Cost of treatment**				
**C14**	Cost of azithromycin (per day, including 10% supply cost)	Mean $0.253	Gamma (α = 29.431, β = 0.009)	[43]
**C15**	Duration of azithromycin	5 days	Uniform (min = 3, max = 7)	Assumption [27–31, 33–35]
**C19**	Cost of amoxicillin (per 5-day course, including 10% supply cost)	Mean $0.848	Gamma (α = 33.913, β = 0.025)	[43]

IgMFA, Immunoglobulin M lateral flow assay; MDR, multi-drug resistant; *S*. Typhi, *Salmonella enterica* serovar Typhi.

A report from five Asian countries showed that the public sector cost of typhoid fever per hospitalised case varied between $0 and $116 (2005) [19]. We assumed that the treatment failure cases would receive additional treatment in a hospital setting after day seven. Therefore, to assess the ICER, the WTP threshold was set at $201 ($116 converted to the year of 2016 using CPI).

## Results

### Incremental cost and health outcomes

The cost, the number of correctly diagnosed typhoid fever cases and the number of treatment success cases in each arm are shown in Table 4. The results of the incremental analyses in CEA are also shown in the same table. In the base-case analysis, the total costs of conducting diagnosis and treatment for 1000 children were $8465 for IgMFA, and $2765 for the clinical diagnosis arm. IgMFA detected 5.87 more true-positives (38.45 true-positive typhoid cases out of 65.17 diseased) per 1000 children than the clinical diagnosis. The cost difference between the two arms was $5700 and the ICER to obtain one additional correct diagnosis of typhoid fever was estimated to be $972. With respect to the primary health outcome, IgMFA had 3.61 more treatment successes than the clinical diagnosis (46.78 versus 43.17). Therefore, the ICER to have one additional treatment success in typhoid fever was estimated to be $1579. We evaluated the impact of starting-up costs on the total cost or to the ICER in CEA. We aimed to compare unit cost of providing a test, and it had a minor impact even if added to the total costs. Thus, the starting-up costs were excluded from the total costs.

**Table 4:** Incremental health outcomes and costs.

Outcome	IgMFA	Clinical diagnosis	Difference
**Health outcome (per 1000 children)**			
Total TyF cases (true-positive and false-negative)	65.17	65.17	-
True-positive cases treated with azithromycin (correctly diagnosed TyF cases)	38.45	32.59	5.87
Treatment success among TyF cases	46.78 (71.8% of total TyF)	43.17 (66.1% of total TyF)	3.61
False-negatives treated with amoxicillin (missed cases)	26.72	32.59	-5.87
False-positives treated with azithromycin (over-treated cases)	18.70	130.88	-112.18
True-negatives treated with amoxicillin	916.13	803.95	112.18
**Cost (per 1000 children)**			
Total cost (not inclusive of start-up costs)	$8465	$2765	$5700
Cost of diagnosis	$7888	$2151	$5737
Cost of treatment	$577	$614	-$37
**Incremental analysis**			
ICER (effect: number of correctly diagnosed TyF)	$972/ correct TyF diagnosis		
ICER (effect: number of treatment success)	$1579/ TyF treatment success		
Cost/child (C/E)	$8.47	$2.76	$5.71
Cost/correct diagnosis TyF (C/E)	$220	$85	$135
Cost/TyF treatment success (C/E)	$181	$64	$117

ICER, Incremental Cost-effectiveness Ratio; TyF, Typhoid fever. C/E (Cost-effectiveness Ratio) was derived from costs/produced health effects. ICER was derived from (Cost of IgMFA-Cost of clinical diagnosis)/ (Effect of IgMFA-Effect of clinical diagnosis).

### Sensitivity analysis

Various one-way sensitivity analyses showed that the ICER to gain one additional treatment success in typhoid fever was sensitive to a change in the sensitivity of both IgMFA and the clinical diagnosis, cost of IgMFA and the prevalence of typhoid fever or MDR strains. The ICER showed mild robustness to the change in treatment effect of amoxicillin and staff costs. The ICER was robust to the effect of a change in the specificity of both tests, treatment effect of azithromycin, and the cost of both azithromycin and amoxicillin.

Table 5 shows the effect of a change in the sensitivity of IgMFA on the difference in health outcomes, cost effectiveness ratio and ICER. The results of one-way sensitivity analyses, changing the sensitivity of the clinical diagnosis, prevalence of typhoid fever and MDR strains are also shown in Table 5. When the sensitivity of IgMFA was changed to 42% (lower 95% CI value), the ICER was estimated to be -$1776 since IgMFA resulted in 3.21 less cases of treatment success than the clinical diagnosis. The ICER would decrease to $285 under a situation of a perfect sensitivity with 20.05 more treatment successful cases. The cost of using IgMFA was still $70 higher per treatment success than the clinical diagnosis.

**Table 5:** One-way sensitivity analysis showing effect of varying sensitivity of IgMFA and clinical diagnosis, and prevalence of typhoid fever or MDR strains (presented in US$, 2016).

	Difference in the number of correctly diagnosed TyF cases (n)	Difference in the number of successfully treated TyF cases (n)	Difference in cost/treatment success (C/E) ($)	ICER ($/treatment success)
**Sensitivity of IgMFA**				
42% (lower 95% CI)	-5.21	-3.21	148	-1776
59% (base-case)	5.87	3.61	117	1579
77% (upper 95% CI)	17.60	10.83	93	527
100% (best-case)	32.59	20.05	70	285
**Sensitivity of clinical diagnosis**				
0% (worst-case)	38.45	23.66	62	241
33% (lower 95% CI)	16.95	10.43	105	547
50% (base-case)	5.87	3.61	117	1579
67% (upper 95% CI)	-5.21	-3.21	126	-1776
**Prevalence of typhoid fever**				
4.5% (lower 95% CI)	4.04	2.49	170	2292
6.5% (base-case)	5.87	3.61	117	1579
9.0% (upper 95% CI)	8.08	4.97	85	1147
**Prevalence of MDR strains**				
25% (best-case)	5.87	2.57	108	2219
50% (base-case)	5.87	3.61	117	1579
90% (worst-case)	5.87	5.27	134	1081

95% CI, 95% confidence interval; ICER, Incremental Cost-effectiveness Ratio; TyF, Typhoid fever. C/E (Cost-effectiveness Ratio) was derived from (costs)/(produced health effects). Difference was derived from (Effect or cost or C/E of IgMFA)-(Effect or cost or C/E of clinical diagnosis). ICER was derived from (Cost of IgMFA-Cost of clinical diagnosis)/(Effect of IgMFA-Effect of clinical diagnosis).

Regarding the effect of the change in the sensitivity of the clinical diagnosis, a 0% of sensitivity showed that the ICER would decrease to $241.

**Fig 2:**
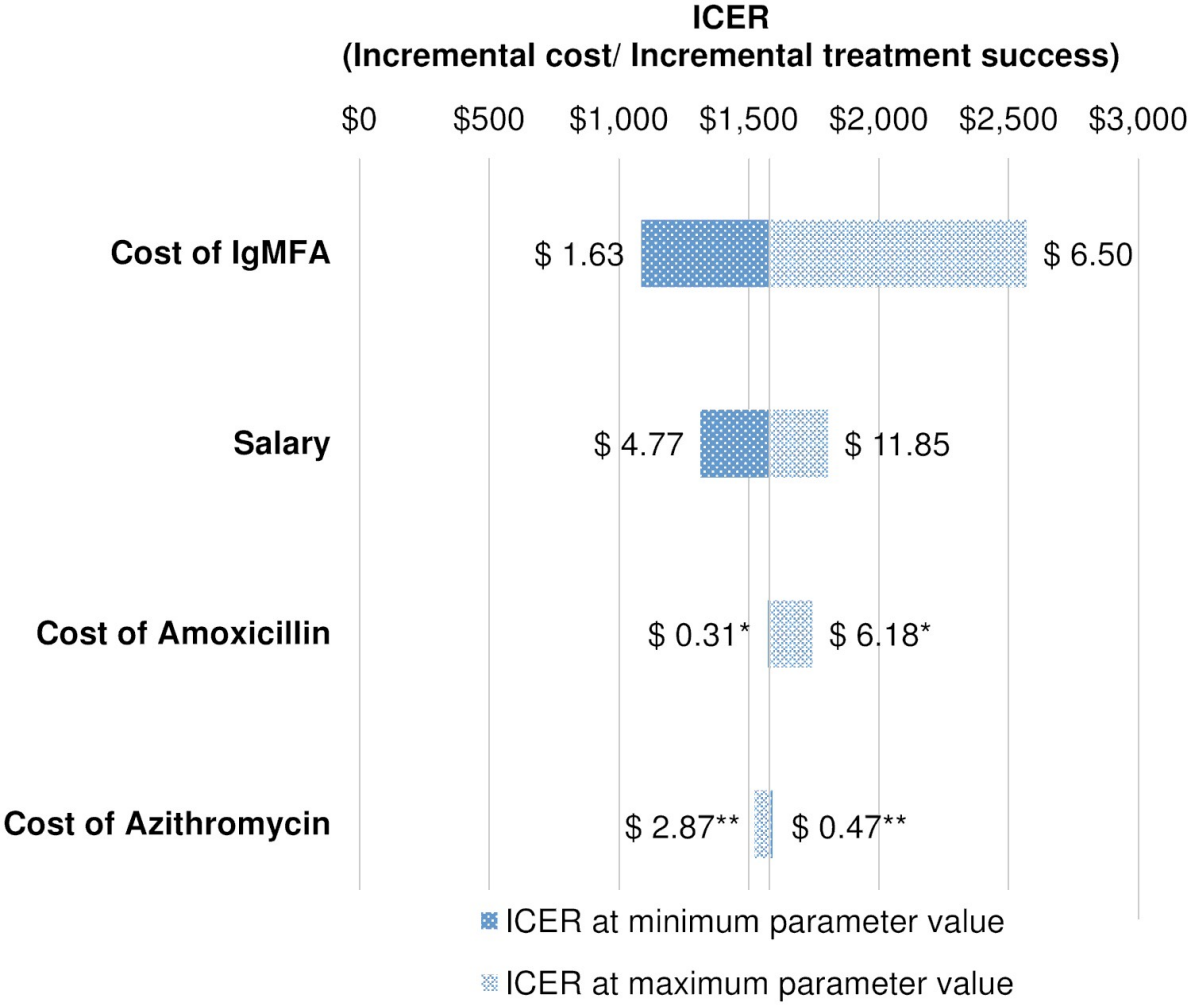
Tornado diagram of ICER change by varying cost parameters. *Cost of amoxicillin is a total cost of 1500mg/day, 5 days course, including 10% supply cost. **Cost of azithromycin is a total cost of 250mg/day, 5 days course, including 10% supply cost. ICER, Incremental Cost-effectiveness Ratio.

At a typhoid fever prevalence of 4.5% (lower 95% CI value), the ICER was estimated to be $2292, while at a prevalence of 9.0% (upper 95% CI value), the estimation was $1147. With a prevalence of MDR strains at 25%, the ICER increased to $2219. With 90% prevalence of MDR strains, IgMFA was more cost-effective ($1081 per an additional treatment success). The change in the specificity of both tests had a minor effect on the ICER (S5 Appendix). The ICER showed a modest change by a change in the probabilities of treatment success in azithromycin and amoxicillin (S5 Appendix). A tornado diagram in Fig 2 presents the effect of changing cost parameters. The ICER was sensitive to the change in the cost of IgMFA. If the cost of IgMFA was reduced to a half price of the base-case ($1.63), the ICER decreased to $1086, while doubling the price ($6.5) resulted in the ICER reaching $2570. A change in salary had a modest effect on the ICER. When the salary was reduced to the lowest value of $4.77 per hour, the ICER decreased to $1314, while the ICER increased to $1804 at the highest salary of $11.85. On the contrary, little effect was seen on the cost-effectiveness of IgMFA when the price of azithromycin or amoxicillin was changed (Fig 2).

**Fig 3:**
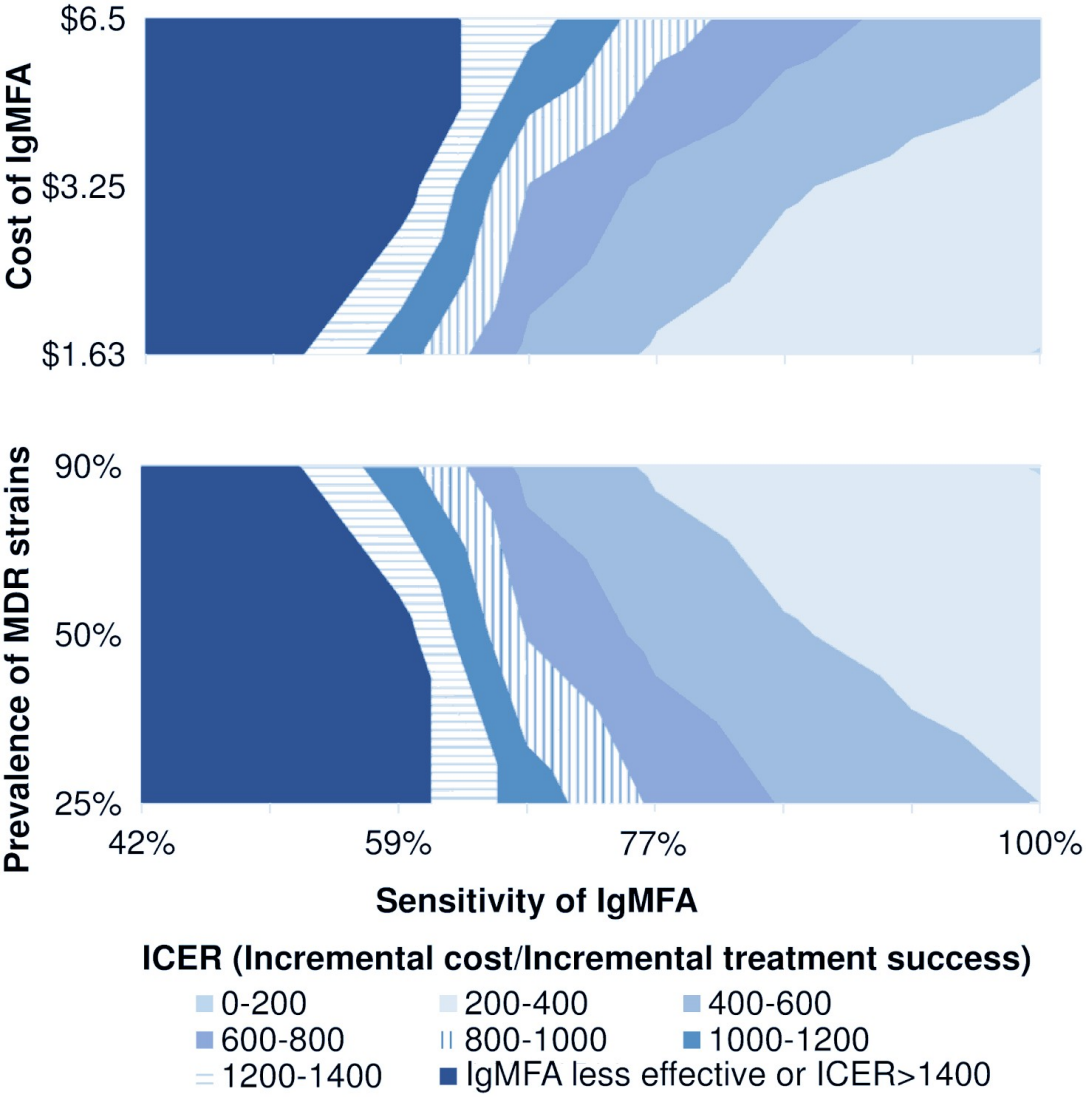
ICER distributions in two-way sensitivity analyses.

Two-way sensitivity analyses were conducted to identify a combination of test characteristics which would fall below a WTP threshold of $201. From the results of the one-way sensitivity analyses, two-way sensitivity analyses were conducted in combinations of parameters as follows: sensitivity of IgMFA and cost of IgMFA, sensitivity of IgMFA and prevalence (both typhoid fever and MDR strains), and cost of IgMFA and prevalence (both typhoid fever and MDR strains). When the sensitivity of IgMFA approached to 100% and the cost of IgMFA was $1.63, the ICER dropped below $201. Distributions of the ICER are shown in Fig 3. When the sensitivity of IgMFA was 100% and the prevalence of MDR strains was 90% (maximum assumed value), the ICER reached $195 (Fig 3). No combination of the sensitivity of IgMFA and the prevalence of typhoid fever, the cost of IgMFA and the prevalence of typhoid fever or MDR strains reached the ICER below $201 (S6 Appendix).

**Fig 4:**
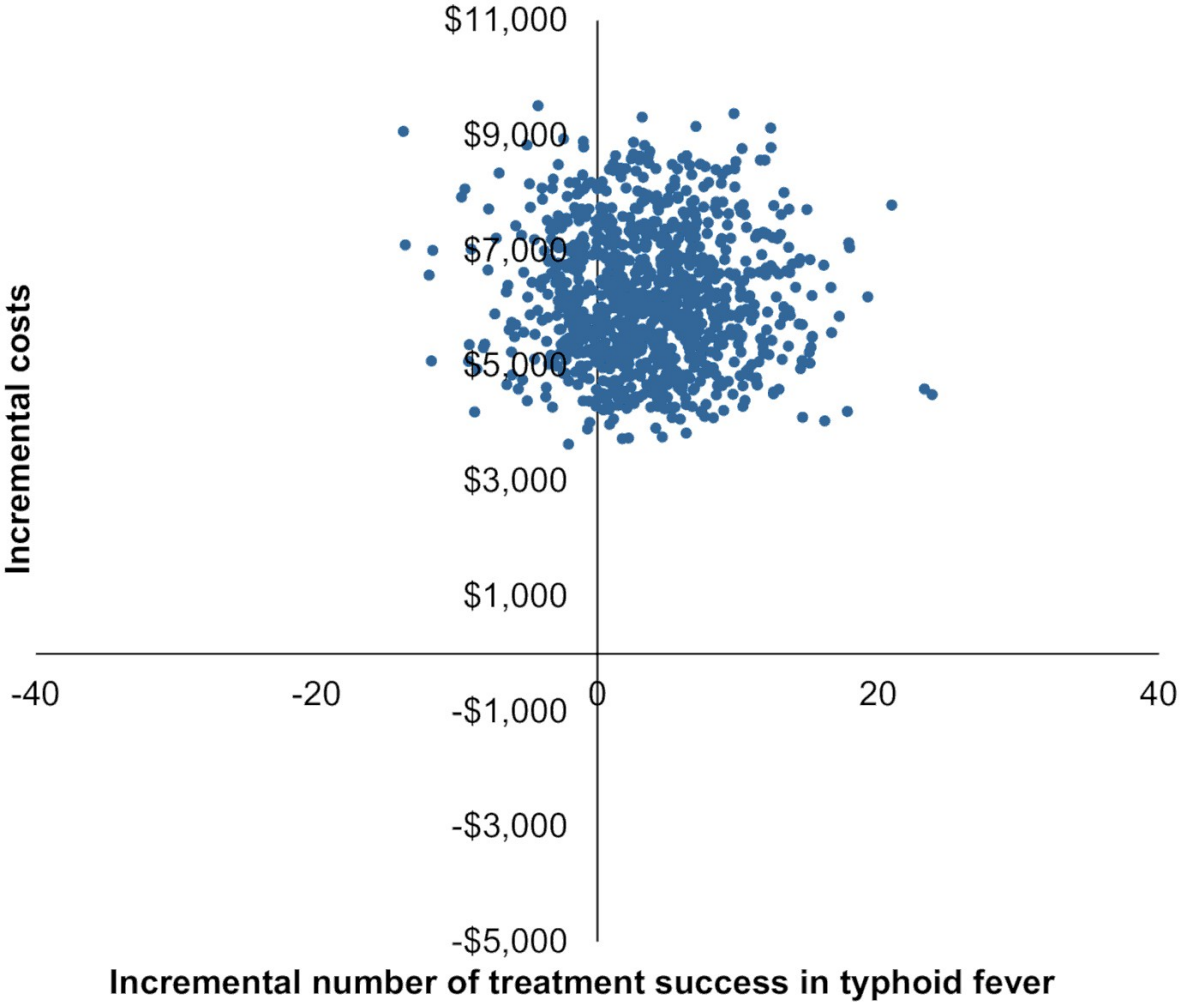
Cost-effectiveness plane in incremental number of treatment success and incremental cost.

Fig 4 shows the results of the probabilistic sensitivity analysis of the cost-effectiveness of replacing clinical diagnosis by IgMFA in terms of a cost-effectiveness plane. Each dot represents a pair of incremental effect and incremental cost, calculated by a combination of random values for parameters, which are assumed to be distributed as per Table 3. The horizontal axis measures incremental number of successfully treated cases when clinical diagnosis was replaced by IgMFA. The vertical axis measures incremental costs when clinical diagnosis was replaced by IgMFA. In all 1000 simulations the cost of IgMFA was higher than that of clinical diagnosis. Most cost-effect pairs lay in the north-east quadrant, suggesting that IgMFA resulted in a larger number of treatment success but was more costly than clinical diagnosis in those pairs. Some cost-effect pairs also lay in the north-west quadrant, meaning the use of IgMFA was less effective and more costly to gain treatment success.

**Fig 5:**
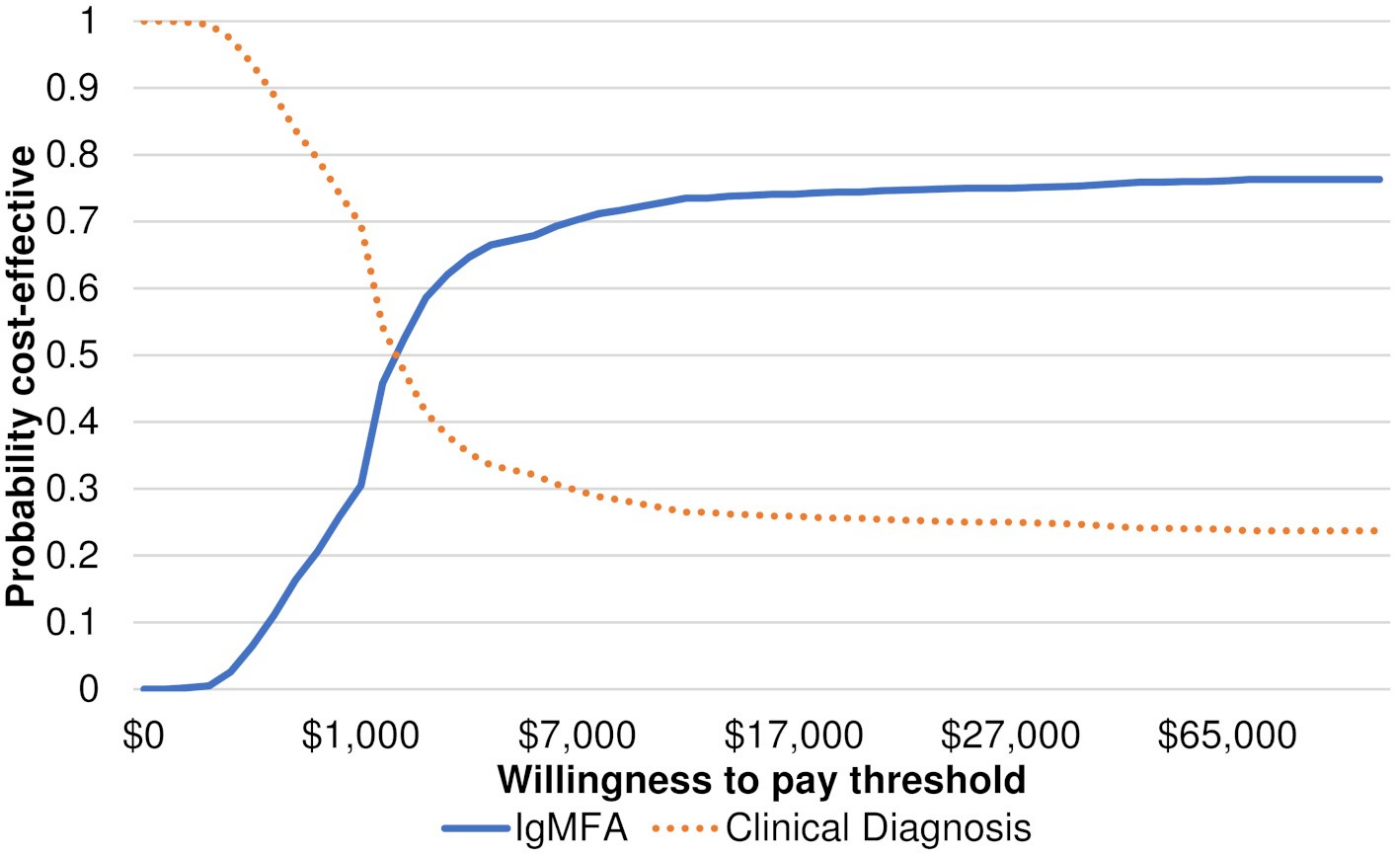
Cost-effectiveness acceptability curve.

A cost-effectiveness acceptability curve was generated to show the probabilities of diagnostic tests being considered as cost-effective, according to a WTP threshold by decision-makers to gain one additional treatment success (Fig 5). For instance, if a decision-maker in Cambodia was willing to pay $1000 per one additional treatment success of typhoid fever, there was a 30.5% probability that the use of IgMFA being cost-effective. However, the probability that the use of IgMFA being cost-effective would increase only to approximately 75%, even a decision-maker was willing to pay more than $15000 for an additional treatment success of typhoid fever case. If the WTP threshold was assumed to be $200, the probability of that IgMFA being cost-effective was 0.2%.

## Discussion

### Summary of findings

The particular IgMFA studied, with a sensitivity of 59% and cost of $3.25, was estimated to be more effective but more costly ($972 per one additional correct diagnosis of typhoid fever; $1579 per one additional treatment success in typhoid fever cases) than the clinical diagnosis in the base-case analysis. One-way sensitivity analyses highlighted that the sensitivity and the cost of IgMFA, the prevalence of typhoid fever and MDR strains are the key drivers for the ICER. The PSA suggests that the probability of IgMFA being cost-effective was 0.2% when WTP threshold to gain one additional treatment success was $201. However, two-way sensitivity analyses showed that in some situations under improved sensitivity and cost in IgMFA, the ICER could decrease below $201 (i.e. cost-effective).

### Limitations

There were several limitations in this study. Cost data were derived from literature reviews and websites. A bottom-up approach for costing was used, which is more precise than the step-down approach but more time-consuming and difficult to implement when detailed data are not available [46, 50]. Aggregated cost data were not available for a step-down approach. The study did not consider healthcare worker adherence to the test results or availability of antimicrobial drugs. In studies using malaria RDTs, adherence to test results has not been perfect [24, 51]. Azithromycin availability may be low in health centre settings and affect the number of day seven treatment successes. Treatment effects derived from trials, some of which included adults, and which were not conducted in Cambodia, may not reflect the true situation. Adherence of patients to treatments is a further variable to be assessed.

In this study, we assumed that malaria cases would have been excluded because in most tropical countries, malaria RDTs are widely used and a positive result would usually result in the patient being treated for malaria with no further testing. However, co-infection is possible and difficult to diagnose clinically. If we had not excluded malaria cases from our analysis, we would expect the IgMFA to increase the correct identification of malaria cases co-infected with typhoid. However, there are no data or reason to suggest that this would be any different from malaria uninfected cases. Therefore, this would not affect the results of the cost-effectiveness analysis. Similarly, although our analysis assumed no pre-treatment with antibiotics, we do not expect that including pre-treatment would affect the results of this analysis.

This study applied CEA for a health outcome measured as the number of correctly diagnosed cases and the number of treatment success at day seven. These health outcomes might not capture the overall effects of introducing IgMFA. There is an uncertainty in relationship between diagnosis and health outcomes and a correct diagnosis may not always translate into the overall benefit [52]. Also, this study might be underestimating the benefit of IgMFA because we are not accounting for the potential benefit in terms of the development of antimicrobial resistance through the reduction in the overuse of antibiotics in patients falsely identified as having typhoid. However, this is complicated by the possible shift of prescribed antibiotic classes, which was reported after malaria RDT [53]. Careful interpretation is necessary for a CEA of a diagnostic test and to obtain the overall effect, calculation of DALYs is necessary. Not only DALYs of true-positives and false-negatives (i.e. patients who have typhoid fever) but also DALYs of false-positives and true-negatives (i.e. patients who have other diseases) are required.

If false-positives were treated with azithromycin, the treatment effect for other diseases has to be also considered. Azithromycin will treat respiratory infections, but not urinary tract infections or other serious invasive bacterial infections including meningitis or sepsis due to other Gram-negative bacteria. The new drug may also change DALYs in other diseases. Under-treatment for typhoid fever may impose other issues, such as increased number of children with complicated disease or relapse and prolonged faecal shedding of the organisms leading to enhanced transmission. These effects may not be captured by a DALY of a patient in a static model but need dynamic modelling.

The probability of treatment success following a clinical diagnosis in this model was derived from data in a hospital setting and a clinical diagnosis by doctor with the benefit of additional blood tests (elevation of liver enzymes, low or normal white cell count, and serum glutamic pyruvic transaminase). The accuracy of clinical diagnosis would be lower available in a remote health centre setting with a basic healthcare worker. Thus, the difference in effect between IgMFA and the clinical diagnosis could be higher, and the IgMFA more cost-effective than the base-case estimation.

### Conclusions

Introducing the IgMFA would lead to a small increase in the number of true typhoid fever cases detected, and a small increase in the number of treatment successes but with a high incremental cost ($1579 per an additional treatment success). Sensitivity analyses did not alter the result that the use of IgMFA was more costly than the presumptive management. The number of children successfully treated by replacing clinical diagnosis with IgMFA depends on the sensitivities of IgMFA and clinical diagnosis.

For the RDT to be cost-effective, a more accurate test is needed. For a cost less than $1.65, and a sensitivity close to 100% with a prevalence of MDR strains of 90%, the IgMFA can be cost-effective. Decision-maker may use a WTP threshold also considering the additional cost incurred when a treatment failure arises.

## Supporting information

S1 Appendix Weight calculation. (TIF)

S2 Appendix Sensitivity and specificity. (TIF)

S3 Appendix Probability of treatment success. (TIF)

S4 Appendix Supplier costs (excluding 10% of shipping cost) US$, 2015. (PDF)

S5 Appendix Effect of varying specificity of tests and probability of treatment success. (TIF)

S6 Appendix ICER change by two-way sensitivity analysis. (TIF)
