# Supplementary figures and images for: Modelling the cost-effectiveness of a rapid diagnostic test (IgMFA) for uncomplicated typhoid fever in Cambodia
Source: PLoS Negl Trop Dis. 2018 Nov 19;12(11):e0006961. doi: 10.1371/journal.pntd.0006961 (PMC6277117; doi:10.1371/journal.pntd.0006961)

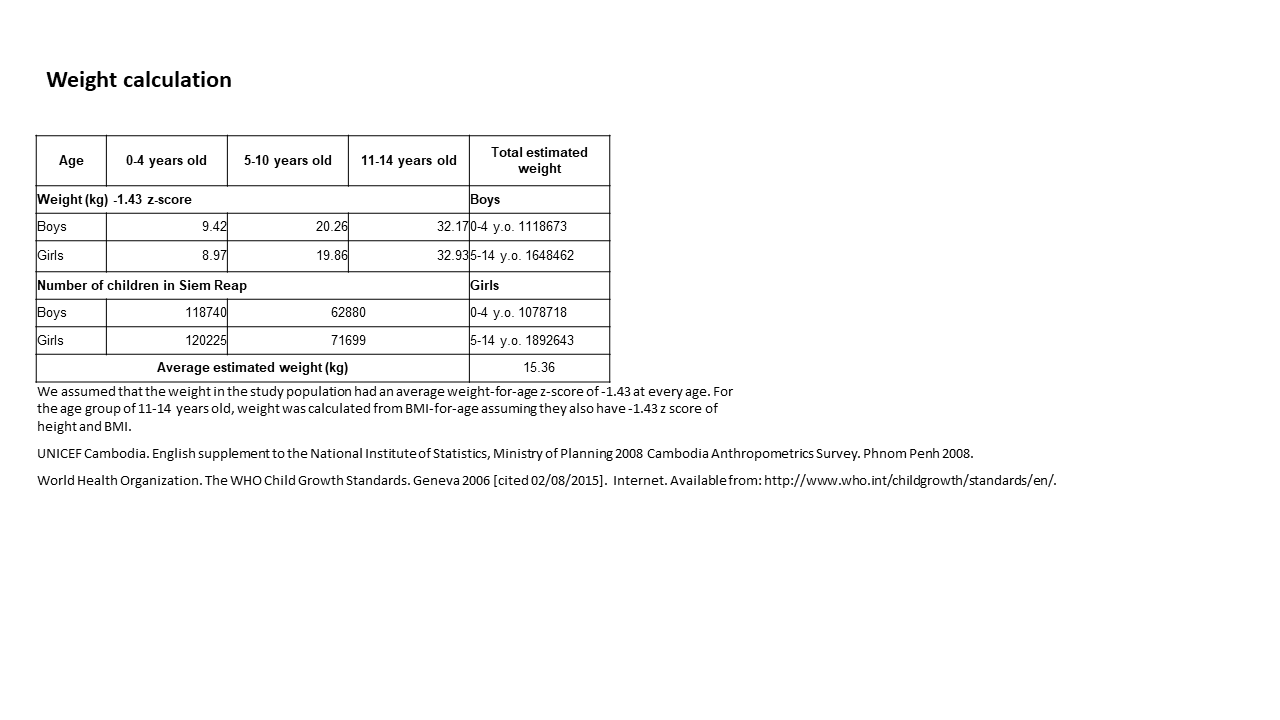

Supplement: S1 Appendix — (TIF) [file pntd.0006961.s001.tif]

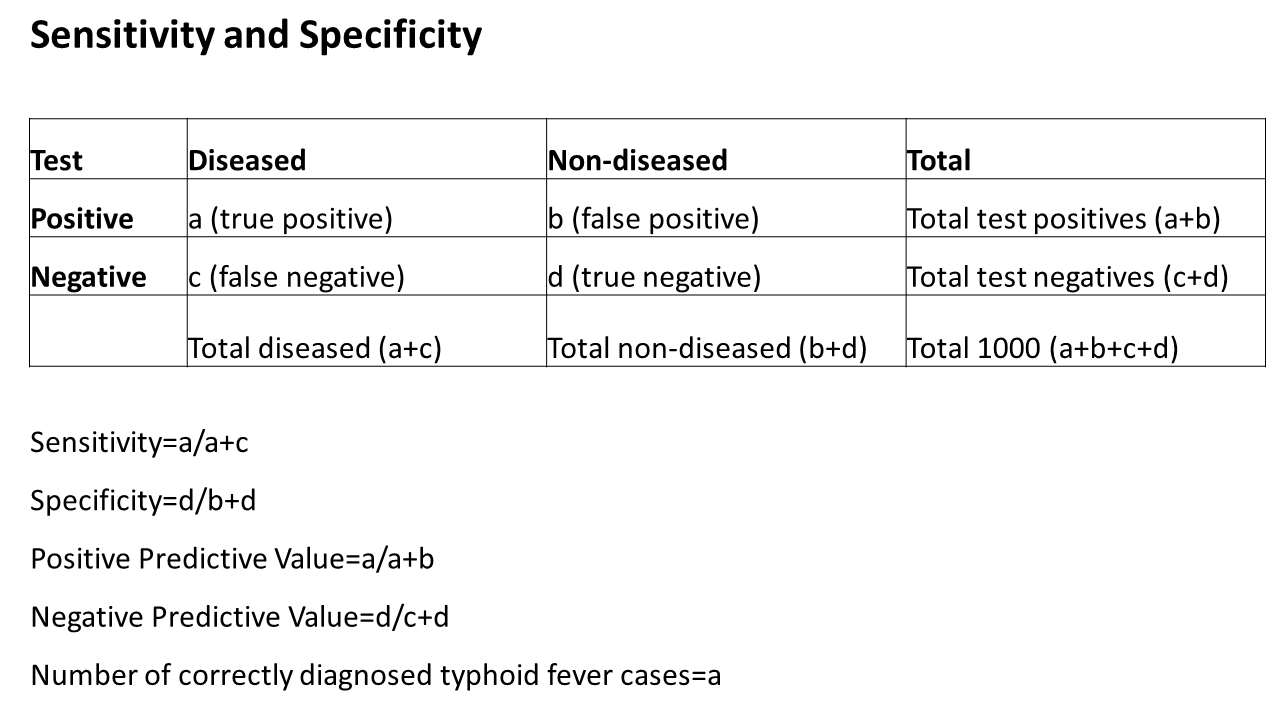

Supplement: S2 Appendix — (TIF) [file pntd.0006961.s002.tif]

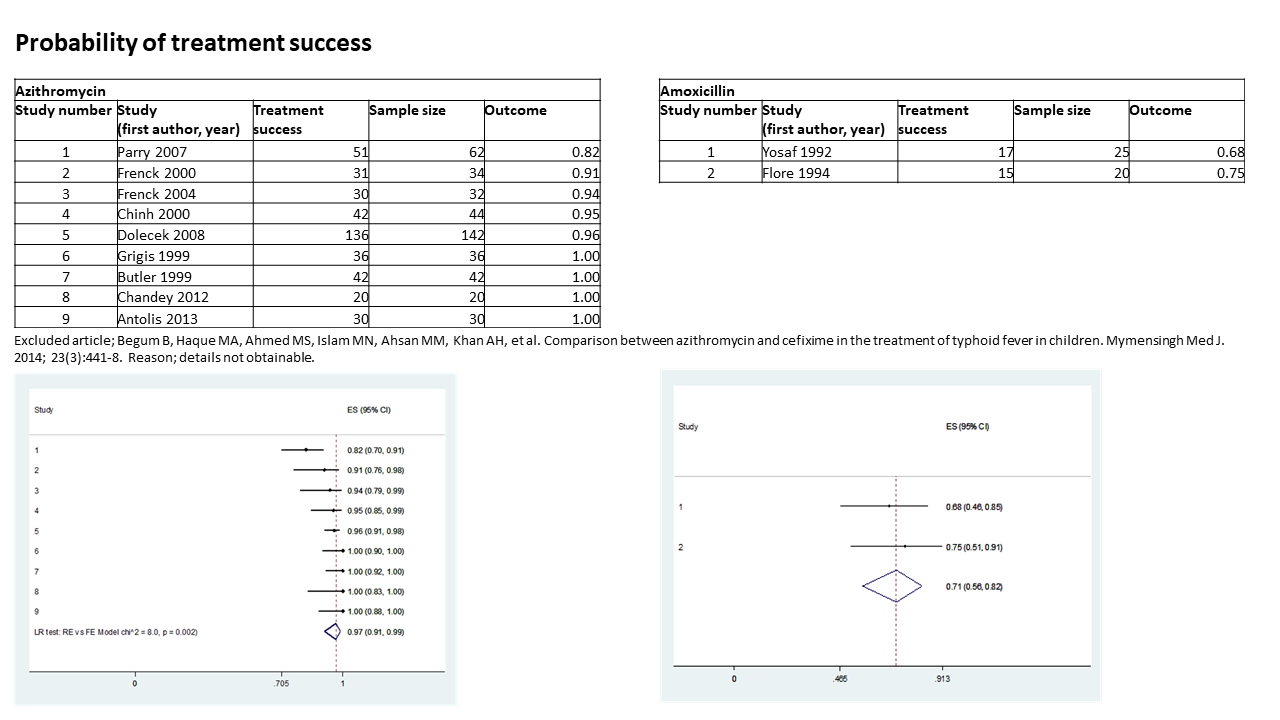

Supplement: S3 Appendix — (TIF) [file pntd.0006961.s003.tif]

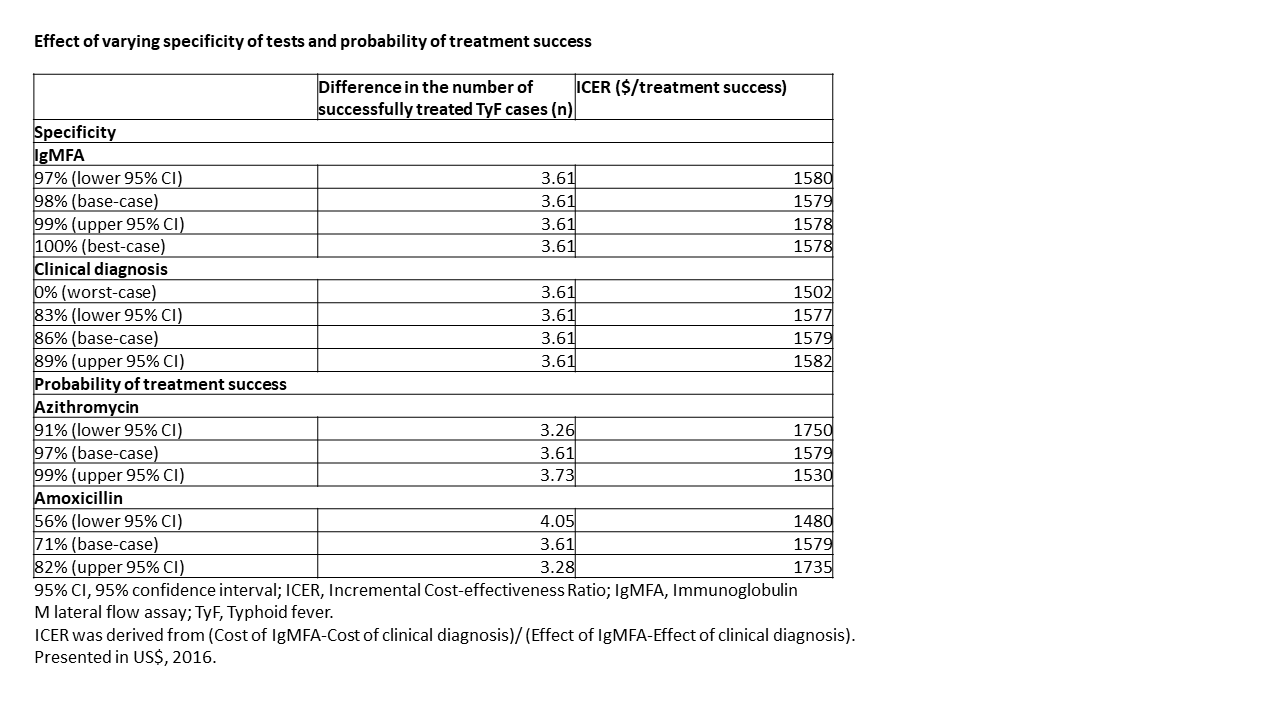

Supplement: S5 Appendix — (TIF) [file pntd.0006961.s005.tif]

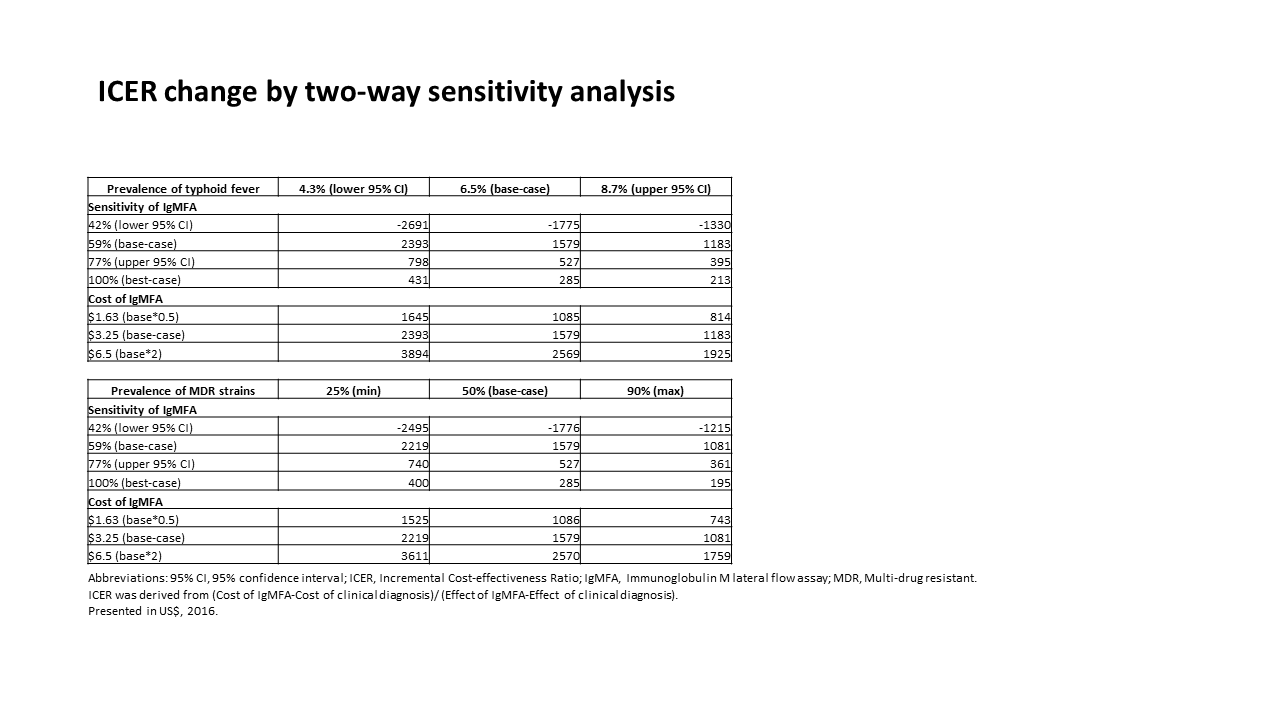

Supplement: S6 Appendix — (TIF) [file pntd.0006961.s006.tif]
