# Supplementary material for: Modelling the cost-effectiveness of a rapid diagnostic test (IgMFA) for uncomplicated typhoid fever in Cambodia
Source: PLoS Negl Trop Dis. 2018 Nov 19;12(11):e0006961. doi: 10.1371/journal.pntd.0006961 (PMC6277117; doi:10.1371/journal.pntd.0006961)
Supplement: S4 Appendix — (PDF) [file pntd.0006961.s004.pdf]

Supplier costs (excluding 10% of shipping cost) US$, 2015

**Azithromycin**			
**Supplier**	**Units**	**Strength**	**US$ per unit**
MEDS	1 Bott(15 ml)	200 mg/5 ml	$ 0.55
UNICEF	1 Bott(30 ml)	200 mg/5 ml	$ 1.45
IMRES	1 Bott(15 ml)	200 mg/5 ml	$ 0.90
UNFPA	1 Bott(15 ml)	200 mg/5 ml	$ 0.99
IDA	120 Bott(15 ml)	200 mg/5 ml	$ 145.92
MEG	100 Tab-cap	250 mg	$ 8.21
UNFPA	6 Tab-cap	250 mg	$ 0.60
MEDS	6 Tab-cap	250 mg	$ 0.64
UNICEF	6 Tab-cap	250 mg	$ 0.70
ASRAMES	6 Tab-cap	250 mg	$ 0.78
IDA	6 Tab-cap	250 mg	$ 0.79
IMRES	4 Tab-cap	250 mg	$ 0.75

**Amoxicillin**			
**Supplier**	**Units**	**Strength**	**US$ per unit**
MISSION	1000 Tab-cap	500mg/tab	$ 18.40
IDA	1000 Tab-cap	500mg/tab	$ 25.09
IMRES	1000 Tab-cap	500mg/tab	$ 27.50
UNICEF	100 Tab-cap	500mg/tab	$ 2.92
MEG	1000 Tab-cap	500mg/tab	$ 29.29
MEDEOR/EU	1000 Tab-cap	500mg/tab	$ 29.78
DURBIN	1000 Tab-cap	500mg/tab	$ 30.06
MEDS	500 Tab-cap	500mg/tab	$ 15.10
ASRAMES	1000 Tab-cap	500mg/tab	$ 30.92
UNFPA	1000 Tab-cap	500mg/tab	$ 36.75
JMS	100 Tab-cap	500mg/tab	$ 4.07
AMSTELFAR	500 Tab-cap	500mg/tab	$ 21.75
UNICEFUNICEF Supply Division(UNICEF)	100 Tab-cap	500mg/tab	$ 4.60
MISSION	1 Bott(100 ml)	250mg/5ml	$ 0.51
MEG	10 Bott(100 ml)	250mg/5ml	$ 5.94
ASRAMES	10 Bott(100 ml)	250mg/5ml	$ 6.10
IMRES	10 Bott(100 ml)	250mg/5ml	$ 6.80
IDA	40 Bott(100 ml)	250mg/5ml	$ 28.29
MEDS	1 Bott(100 ml)	250mg/5ml	$ 0.80
UNFPA	40 Bott(100 ml)	250mg/5ml	$ 36.30
AMSTELFAR	1 Bott(100 ml)	250mg/5ml	$ 0.97
MISSION	1000 Tab-cap	250mg/tab	$ 11.89
MSD/TANZ	1000 Tab-cap	250mg/tab	$ 12.52
UNICEF	1000 Tab-cap	250mg/tab	$ 13.90
MEDEOR/TZ	1000 Tab-cap	250mg/tab	$ 14.60
MEDEOR/TZ	100 Tab-cap	250mg/tab	$ 1.52
MEDS	1000 Tab-cap	250mg/tab	$ 15.30
ASRAMES	1000 Tab-cap	250mg/tab	$ 15.62
IMRES	1000 Tab-cap	250mg/tab	$ 15.75
MEG	1000 Tab-cap	250mg/tab	$ 16.14
IDA	1000 Tab-cap	250mg/tab	$ 16.31

IDA	1000 Tab-cap	250mg/tab	$ 16.43
MEDEOR/EU	1000 Tab-cap	250mg/tab	$ 16.40
DURBIN	1000 Tab-cap	250mg/tab	$ 17.96
JMS	100 Tab-cap	250mg/tab	$ 1.93
UNFPA	1000 Tab-cap	250mg/tab	$ 23.06
AMSTELFAR	1000 Tab-cap	250mg/tab	$ 23.20
UNICEF	100 Tab-cap	250mg/tab	$ 2.28
IDA	100 Tab-cap	250mg/tab	$ 3.15
MISSION	260 Tab-cap	250mg/tab	$ 47.26
